# Integrated Analysis of the Metabolome and Transcriptome on Anthocyanin Biosynthesis in Four Developmental Stages of *Cerasus humilis* Peel Coloration

**DOI:** 10.3390/ijms222111880

**Published:** 2021-11-02

**Authors:** Xiaolong Ji, Jing Ren, Yixin Zhang, Shaoyu Lang, Di Wang, Xingshun Song

**Affiliations:** 1 Key Laboratory of Saline-alkali Vegetation Ecology Restoration (Northeast Forestry University), Ministry of Education, Harbin 150040, China; jxl18019880632@gmail.com (X.J.); zyx9071@nefu.edu.cn (Y.Z.); langshaoyu95@gmail.com (S.L.); wd15033395126@gmail.com (D.W.); 2 Department of Genetics, College of Life Science, Northeast Forestry University, Harbin 150040, China; 3 College of Food Science, Key Laboratory of Dairy Science, Ministry of Education, Northeast Agricultural University, Harbin 150030, China; rj15945688512@gmail.com

**Keywords:** *Cerasus humilis*, anthocyanin, pigmentation, transcriptome, metabolites

## Abstract

*Cerasus humilis* is a unique dwarf shrub and fruit color is an important trait in the species. In this study, we evaluated the transcriptomic and metabolomic profiles of the plant at different developmental stages to elucidate the mechanism underlying color formation. In a metabolomics analysis, 16 anthocyanin components were identified at four developmental stages, and high levels of cyanidin *O*-syringic acid and pelargonidin 3-*O*-beta-d-glucoside (callitephin chloride) were correlated with the reddening of the fruit peel. A co-expression analysis revealed that *ANS* and *UFGT* play key roles in pigmentation (PCC > 0.82). Additionally, transcriptome data showed that most anthocyanin biosynthetic genes and two MYB transcription factors were significantly up-regulated. QRT-PCR results for these differentially expressed genes were generally consistent with the high-throughput sequencing. Moreover, the overexpression of *ChMYB1* (TRINITY_DN21536_c0_g1) in apple calli could contribute to the accumulation of anthocyanin. It was also found that *UFGT* (TRINITY_DN19893_c1_g5) and *ChMYB1* (TRINITY_DN21536_c0_g1) have similar expression patterns. These findings provide insight into the mechanisms underlying anthocyanin accumulation and coloration during fruit peel development, providing a basis for the breeding of anthocyanin-rich *C. humilis* cultivars.

## 1. Introduction

*Cerasus humilis* (Bge.) Sok.(2n = 2x = 16), which belongs to the genus *Cerasus* in the family Rosaceae, is a small dwarf shrub that originated from China [1]. It is highly stress-resistant, especially to drought and low temperatures, which is an excellent wind-breaking and sand-fixing shrub species [2,3,4]. The fruit peel of *C. humilis* have a bright color and show a clustered arrangement, they are known as “calcium fruits” owing to their relatively high calcium contents [5]. The fruits are rich in flavonoids, including anthocyanins, proanthocyanidins, organic acids, and polyphenols [6,7,8]. *C. humilis* seeds (semen pruni) have been used as a traditional Chinese medicine for heat-clearing and anti-water swelling [9]. Based on its ecological and economic benefits, *C. humilis* has become an emerging multipurpose fruit tree in the last 10 years, with a total estimated planting area of approximately 20,000 ha [7,8].

Anthocyanins belong to the flavonoid class of phenolic compounds [10]. They are a group of water-soluble pigments in plants that contribute to fruit development, tolerance to environmental stresses, and human health [7,11,12]. Genes involved in anthocyanin biosynthesis have been well-characterized in many plants, including *Arabidopsis thaliana* [13], apple (*Malus pumila*) [14], tomato (*Solanum lycopersicum*) [15], and pear (*Pyrus pyrifolia*) [16]. Briefly, anthocyanins are synthesized from phenylalanine via the phenylpropanoid and flavonoid pathways in the cytoplasm [12]. Many functional genes, including genes encoding phenylalanine ammonia-lyase (*PAL*) [17], cinnamic acid 4-hydroxylase (*C4H*) [18], 4-coumarate-CoA ligase (*4CL*) [19], chalcone synthase (*CHS*) [20], chalcone isomerase (*CHI*) [21], flavanone 3-hydroxylase (*F3H*) [22], dihydroflavonol 4-reductase (*DFR*) [23], anthocyanidin synthase (*ANS*; also called leucoanthocyanidin dioxygenase [*LDOX*]) [24], and UDP-glucose: flavonoid-3-*O*-glycosyltranferase (*UFGT*) [25], play important roles in the production of anthocyanins. Furthermore, MYB transcription factors (TFs) are key factors in regulatory networks controlling growth and development, metabolism, and responses to biotic and abiotic stresses [26]. For example, anthocyanin accumulation is regulated by the MYB transcription factor [10,27,28,29]. Flavonoid biosynthesis, controlled by a diverse array of exogenous and endogenous factors, is the most well-studied secondary metabolic pathway in plants, including *Actinidia*, in which anthocyanins are the end product of the flavonoid metabolic pathway [30]. Gene expression controlling the accumulation of metabolites under different stages of development is a direct determinant of fruit color changes. However, the molecular and metabolic pathways involved in *C. humilis* fruit peel coloration are still elusive. Therefore, the genetic basis of anthocyanin biosynthesis and related metabolic pathways requires further exploration. Knowledge of the genetic basis of characters related to fruit coloration will facilitate the manipulation of this trait to obtain more attractive and healthier fruits for consumers [31].

Transcriptomics and metabolomics can provide insight into transcriptional regulation and metabolic flow. Bai et al. [32] performed a spatiotemporal transcriptomic analysis of the red Chinese sand pear peels, revealing that a light-responsive pathway functions in anthocyanin accumulation with different temporal expression patterns, they suggested that *PpMYB10* as well as other light-responsive transcription factors are involved in the regulation of anthocyanin biosynthesis. Using a combination of transcriptomic and metabolite profiling, Zhuang et al. [33] found that purple turnip essentially diverts dihydroflavonols to the biosynthesis of anthocyanins over flavonols by strongly down-regulating one flavonol synthase gene, while strikingly up-regulating dihydroflavonol 4-reductase, anthocyanin synthase, and UDP-glucose: flavonoid-3-*O*-glucosyltransferase compared to levels in green turnip. In the same way, Xue et al. [34] found that *CHS* gene, *F3′H* genes, and *DFR* genes may be the key functional genes controlling the formation of pink and red testa in peanut by network analyses of metabolomics and transcriptomics data. Thus, multi-omics joint analysis is a powerful means to elucidate the complex process of plant development [10,35].

In the present study, based on UPLC-MS/MS, metabolite changes in the process of peel color change in *C. humilis* were qualitatively and quantitatively analyzed, and the unique pattern of anthocyanin-related metabolites during peel color change was established for the first time. Additionally, a correlation analysis of the transcriptomic profiles and anthocyanin contents was performed to elucidate the mechanism underlying variation in fruit peel pigmentation in *C. humilis*, followed by further analyses of the regulatory mechanism based on gene expression assays. Sixteen anthocyanin components were identified at four developmental stages, and high levels of cyanidin *O*-syringic acid and pelargonidin 3-*O*-beta-d-glucoside (callitephin chloride) were correlated with the reddening of the fruit peel. A co-expression analysis revealed that *ANS* and *UFGT* play keys role in pigmentation. Herein, we also showed that the overexpression of *ChMYB1* (TRINITY_DN21536_c0_g1) in apple calli could contribute to the accumulation of anthocyanin. These findings provide a potential biotechnological strategy for the breeding of anthocyanin-rich *C. humilis* cultivars.

## 2. Results

### 2.1. Principal Component Analysis (PCA) Reveals Differences in Anthocyanin Metabolite Profiles

**Figure 1:**
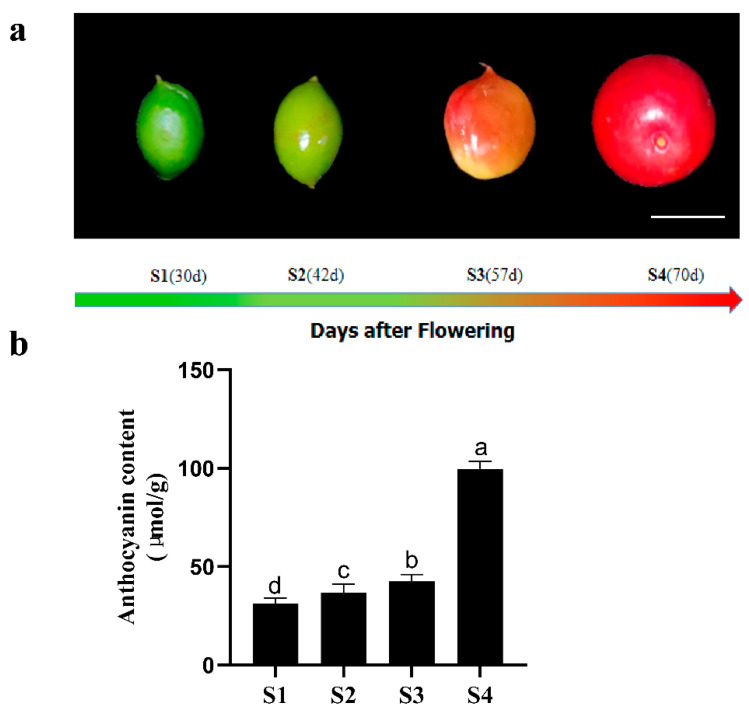
Pigmentation and phenotypes of *Cerasus humilis* fruit peel during four stages. (**a**) fruit phenotypes: young fruit (S1), green fruit (S2), slightly red fruit (S3), and red fruit (S4). (**b**) anthocyanin content of four stages of *Cerasus humilis* fruit peel. Scale bar, 1 cm. Lowercase letters indicate significant differences at *p* < 0.05.

**Figure 2:**
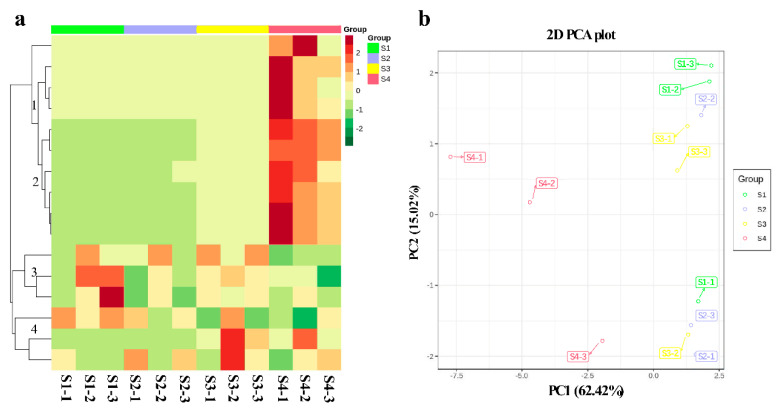
Heat map and PCA of the relative differences in anthocyanins in different fruit development stages in *Cerasus humilis* fruit peel; (**a**) Heat map visualization. Each sample is visualized in a single column, and each metabolite is represented by a single row. Red indicates high abundance and green indicates low abundance; (**b**) Score plots for PCA. Young fruit (S1), 30 days after flowering, green fruit (S2), 42 days after flowering, slightly red fruit (S3), 57 days after flowering, and red fruit (S4), 70 days after flowering. Metabolite names are listed in Table S1.

Traditionally and practically, the fruit peel of *C. humilis* is classified into four developmental stages (Figure 1). Metabolite content data processing was performed to improve normalization using R (www.r-project.org/) which accessed on 12 December 2019, to compare the accumulation of metabolites in different samples by HCA (Hierarchical cluster analysis). Based on relative differences in accumulation patterns among different stages, the HCA of anthocyanins resulted in four main clusters in *C. humilis* fruit peel (Figure 2a). Anthocyanins belonging to clusters 1 and 2 accumulated at the highest levels in S4, while anthocyanins within clusters 3 and 4 did not differ significantly among stages.

Metabolite profiles of the fruit peel were then subjected to a principal component analysis (PCA) [36]. The first two principal components (PC1 and PC2) for the fruit peel explained 62.42% and 15.02% of the total variance, respectively (Figure 2b). The fruit peel contained 16 anthocyanins based on subsequent HPLC-MS and MRM (multiple reaction monitoring) analyses (Supplementary Table S1), with 11 differential expression metabolites (fold change ≥ 2 or fold change ≤ 0.5), including peonidin *O*-hexoside, cyanidin 3-*O*-malonylhexoside, cyanidin *O*-syringic acid, pelargonidin *O*-acetylhexoside, cyanidin 3-*O*-glucoside (kuromanin), procyanidin A1, cyanidin 3,5-*O*-diglucoside (cyanin), pelargonidin 3-*O*-beta-d-glucoside (callitephin chloride), cyanidin 3-*O*-galactoside, and peonidin 3-*O*-glucoside chloride. Apart from procyanidins, the predominant anthocyanins were cyanidin and its glycoside-derived compounds. Among the anthocyanin compounds discovered in fruit peel, two were found at particularly high concentrations in S4, suggesting that these two compounds (cyanidin *O*-syringic acid and pelargonidin 3-*O*-beta-d-glucoside (callitephin chloride)) contribute substantially to the mechanism underlying fruit peel coloration.

### 2.2. Overview of Transcriptome Sequencing

To assess the gene expression profile of the fruit peel at S1–S4, RNAs from four stages of fruit peels were used for RNA-seq, with three replicates per stage. Before using Illumina platform to read the cDNA libraries, the low-quality reads and adapter sequences were deleted. The clean reads for each sample ranged from 40,704,832 to 47,000,394, and a total of 541,781,582 clean reads were obtained. The Q30 values > 92%, meeting the criterion for gene discovery. Based on filtered clean data, the full-length transcript sequence was assembled using Trinity, and the total number of assembled transcripts was 176,109, with a mean length of 1340.05 nt and N50 length of 2227 nt. These were assembled into 73,035 unigenes with a mean length of 1018 nt and N50 length of 1917 nt (Supplementary Table S2).

### 2.3. Annotation and Identification of Unigenes

All assembled unigenes and ORFs were annotated using Trinotate against the public databases NT, NR, UniProt, RNAMMER, eggNOG, GO, and KEGG. According to the GO database, 73,035 unigenes are divided into 60 functional groups, in the three main functional categories (biological process, cellular component, and molecular function) (Supplementary Figure S1). In the biological process category, 42,236 (57.83%) unigenes were associated with metabolic processes.

**Figure 3:**
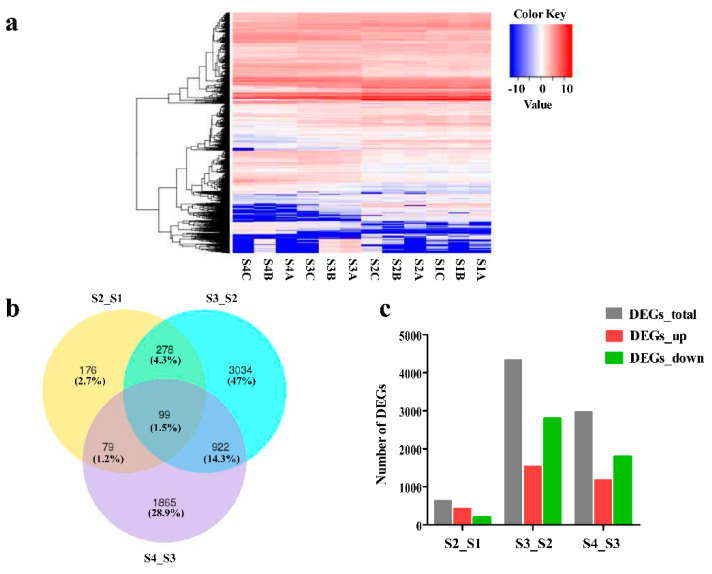
Analysis of transcriptomic data. (**a**) Heat map of DEGs in different developmental stages of *Cerasus humilis* fruit peel; (**b**) Venn diagram of DEGs revealed in pair-wise comparisons; (**c**) The number of DEGs in pair-wise comparisons, |log2 Ratio| ≥ 1 and q < 0.05. Young fruit (S1), 30 days post-anthesis (DPA), green fruit (S2), 42 days post-anthesis (DPA), slightly red fruit (S3), 57 days post-anthesis (DPA), and red fruit (S4), 70 days post-anthesis (DPA). A, B, and C are three biological replicates.

To gain more insight into the transcriptomic differences, we compared DEGs (Differential expression genes) in *C. humilis* fruit peel among different developmental stages. Using the DESeq2 package, normalized RPKM (Reads Per Kilobase Millon Mapped Reads) values were obtained from RNA-seq data to quantify transcript expression. We identified 7930 DEGs in comparisons among different fruit developmental stages. Among the three comparisons (S2_S1, S3_S2, and S4_S3), the largest number of DEGs was found between S3 and S2, with 4333 DEGs, including 1540 up-regulated and 2793 down-regulated DEGs. In addition, there were 430 up-regulated DEGs in S2_S1, and 1179 up-regulated DEGs in S4_S3 (Figure 3; Supplementary Figure S2). These results indicated that DEGs were most active during the second developmental stage of the fruit peel and were perhaps largely responsible for anthocyanin biosynthesis. Therefore, we speculate that the transition from S2 to S3 is a key stage in fruit peel development.

### 2.4. GO Enrichment and KEGG Pathway Analyses of DEGs

We functionally categorized the DEGs based on GO terms. A total of 7927 unigenes were annotated, including 429 up-regulated and 201 down-regulated unigenes in the S2_S1-enriched GO term libraries and 1539 up-regulated and 2794 down-regulated DEGs in the S3_S2-enriched GO term libraries. The subcategories with the highest degree of enrichment were all cell part, followed by binding and cellular process, as shown in a histogram in Figure S3. Apart from this, 1178 up-regulated and 1786 down-regulated unigenes in the S4_S3 comparison were enriched for GO terms, particularly cell part, followed by cellular process, and binding.

To classify the DEGs based on related pathways, a KEGG enrichment analysis was performed. In total, 125 biosynthetic and metabolic pathways were enriched in the pairwise comparisons of fruit peel developmental stages. The flavonoid biosynthetic pathway began to be enriched (ko00941, 10 genes) in S3_S2, and the anthocyanin biosynthetic pathway began to be enriched (ko00942, 2 genes) in S4_S3. We obtained 12 DEGs across the three comparisons related to pigment synthesis, suggesting that these genes are associated with fruit peel coloration (Supplementary Figure S4).

### 2.5. Analysis of Unigenes Related to Anthocyanidin Biosynthetic Pathways in the Fruit Peel

**Figure 4:**
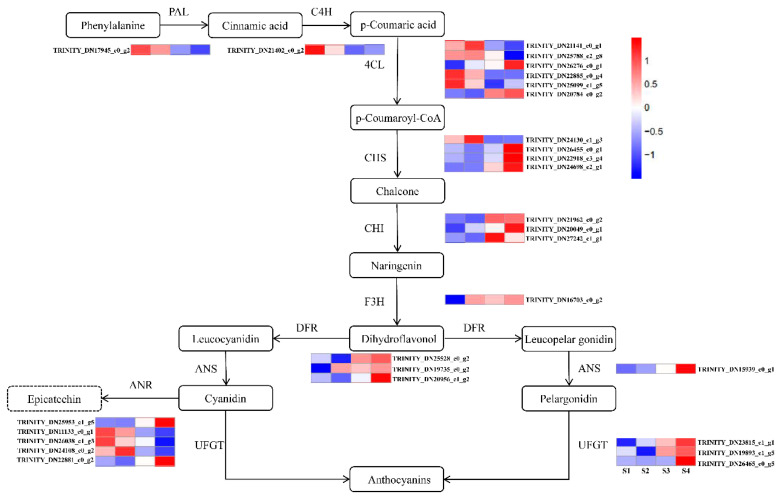
Anthocyanin biosynthetic pathway and their genes expression levels in fruit peel development. Blue and red in the heatmap represent the down- and up-regulated structural genes, respectively. Differentially expressed genes including phenylalanine ammonia-lyase (*PAL*), cinnamate 4-monooxygenase (*C4H*), 4-coumarate-CoA ligase (*4CL*), chalcone synthase (*CHS*), chalcone isomerase (*CHI*), flavanone-3-hydroxylase (*F3H*), dihydroflavonol 4-reductase (*DFR*), anthocyanidin reductase (*ANR*), anthocyanidin synthase (*ANS*) and UDP-glucose: flavonoid-3-*O*-glycosyltranferase (*UFGT*). Four colored boxes showed the relative gene expression level measured by RNA-seq (average value). Gene names are listed in Table S3.

**Figure 5:**
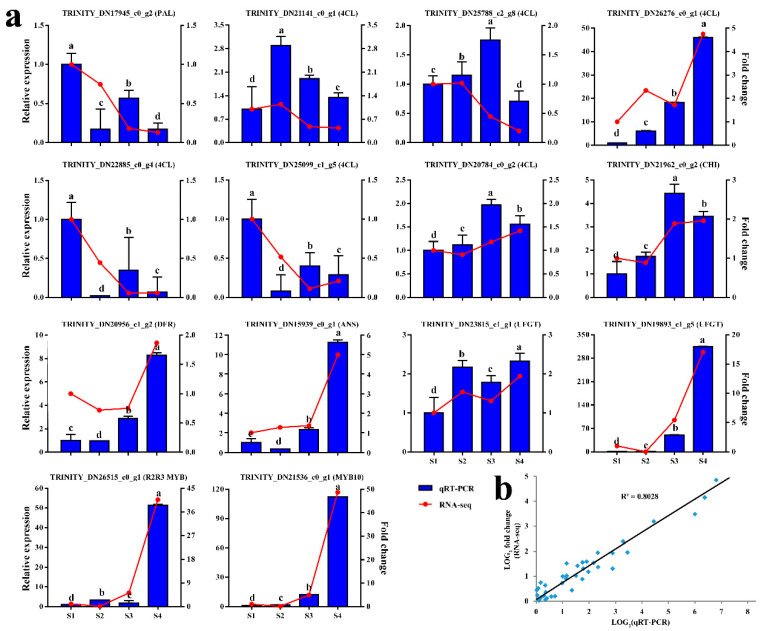
QRT-PCR validation of representative anthocyanin-related genes in fruit peel development. (**a**) The left *y*-axis indicates the corresponding expression data from qRT-PCR (blue histogram). The right *y*-axis shows the relative gene expression level measured by RNA-seq (red lines). *Chactin* was used as reference gene for normalization. All gene expression levels in S1 were set to 1. (**b**) Correlation of the expression levels of 14 selected genes measured by qRT-PCR and RNA-seq values for mean expression and standard errors (SE, *n* = 3) indicated by vertical bars. Lowercase letters indicate significant differences at *p* < 0.05. Gene names including phenylalanine ammonia-lyase (*PAL*), 4-coumarate-CoA ligase (*4CL*), chalcone isomerase (*CHI*), dihydroflavonol 4-reductase (*DFR*), anthocyanidin synthase (*ANS*) and UDP-glucose: flavonoid-3-*O*-glycosyltranferase (*UFGT*).

Previous studies have suggested that the anthocyanin biosynthetic pathway is an important metabolic branch of the flavonoid pathway, responsible for the production of anthocyanins in different plant tissues. Thus, we evaluated the mechanisms underlying red pigmentation in fruit peel based on a comparative transcriptome analysis. The compositions of compounds in anthocyanin biosynthetic pathways differed significantly depending on the growth stage (Figure 4). The *PAL* gene regulates the synthesis of cinnamic acid and consistently showed a gradual decrease over the four growth stages. Seven *4CL* genes exhibited significant differences in expression levels, including 2 up-regulated and 5 down-regulated genes. The *CHS* gene is the first key enzyme in the flavonoid pathway, which catalyzes the synthesis of chalcone; it consistently showed a gradual increase over the four growth stages. The expression levels of *CHI*, *F3H*, *DFR*, and *ANS* were significantly up-regulated from S1 to S4, and the expression levels were positively correlated with the accumulation of anthocyanin. UDP-glucose: flavonoid-3-*O*-glycosyltranferase (*UFGT*), which mainly catalyzes the transformation of unstable anthocyanin glycosides into stable anthocyanin. By using a transcriptomic analysis, two *UFGT* genes (TRINITY_DN23815_c1_g1 and TRINITY_DN19893_c1_g5) involved in the anthocyanin biosynthetic pathway were screened (Figure 4). In order to test the accuracy of the transcriptome data, we selected 12 anthocyanin synthesis genes and 2 MYB genes for qRT-PCR verification (Figure 5a). The Pearson’s correlation coefficient between RNA-seq and qRT-PCR was 0.8028 (Figure 5b). The results showed that the expression trend of qRT-PCR was consistent with the transcriptome data, indicating that the transcriptome data has high reliability.

### 2.6. Integrated Analysis of the Transcriptome and Metabolome

**Figure 6:**
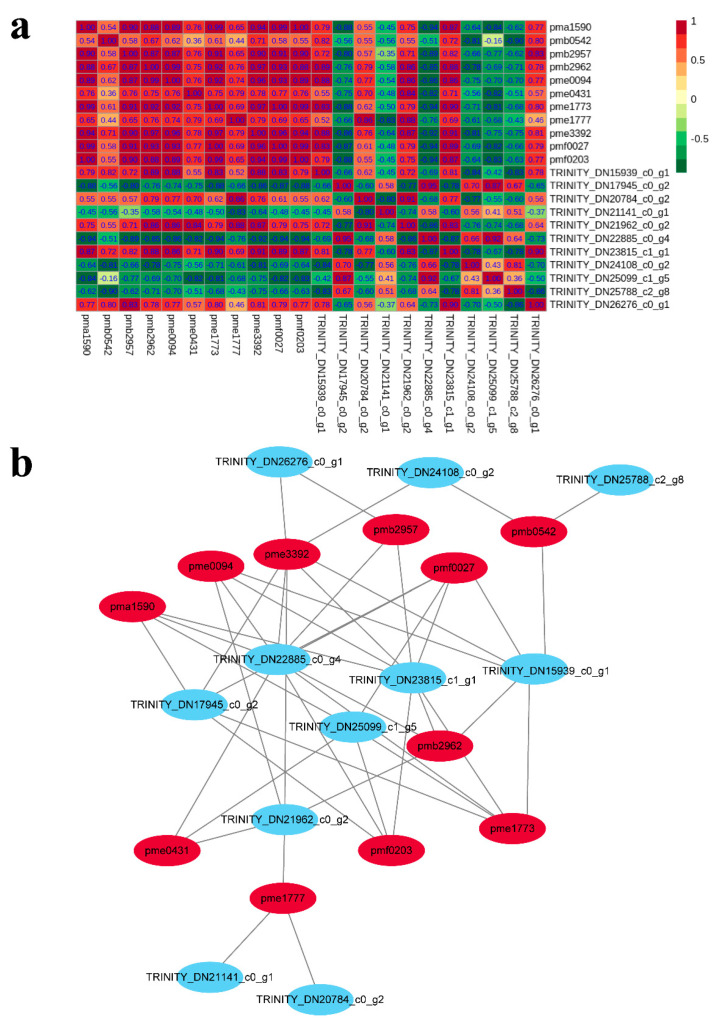
Correlation analysis of metabolites and DEGs. (**a**) Red indicates a positive correlation, green indicates a negative correlation. A deeper color indicates a stronger correlation. (**b**) Results of the correlation analysis between metabolites and DEGs. The networks between metabolites and transcripts were visualized with Cytoscape software. Gene ID including TRINITY_DN15939_c0_g1 (anthocyanidin synthase), TRINITY_DN17945_c0_g2 (phenylalanine ammonia lyase), TRINITY_DN20784_c0_g2 (4-coumarate--CoA ligase), TRINITY_DN21141_c0_g1 (4-coumarate--CoA ligase-like), TRINITY_DN21962_c0_g2 (Chalcone isomerase like), TRINITY_DN22885_c0_g4 (4-coumarate--CoA ligase), TRINITY_DN23815_c1_g1 (UDP-glucose:flavonoid-3-*O*-glycosyltranferase), TRINITY_DN24108_c0_g2 (Putative anthocyanidin reductase), TRINITY_DN25099_c1_g5 (4-coumarate--CoA ligase), TRINITY_DN25788_c2_g8 (4-coumarate--CoA ligase-like) and TRINITY_DN26276_c0_g1 (Oxalate--CoA ligase); Meta ID including pma1590 (Peonidin *O*-hexoside), pmb0542 (Cyanidin 3-*O*-malonylhexoside), pmb2957 (Cyanidin *O*-syringic acid), pmb2962 (Pelargonidin *O*-acetylhexoside), pme0094 (Cyanidin 3-*O*-glucoside (Kuromanin)), pme0431 (Procyanidin A1), pme1773 (Cyanidin 3-*O*-rutinoside (Keracyanin)), pme1777 (Cyanidin 3,5-*O*-diglucoside (Cyanin)), pme3392 (Pelargonidin 3-*O*-beta-d-glucoside (Callitephin chloride)), pmf0027 (Cyanidin 3-*O*-galactoside) and pmf0203 (Peonidin 3-*O*-glucoside chloride). Related gene and metabolites are listed in Table S4 (|PCC| > 0.8).

By transcriptome and metabolome analyses, we constructed a co-expression network to identify interactions (Figure 6). We found that 11 anthocyanin biosynthesis DEGs and 11 differential expression metabolites exhibited interactions. Highly positive correlations were obtained among most differential expression metabolites, and only pmb0542 (cyanidin 3-*O*-malonylhexoside) was minimally relevant to all other differential expression metabolites. The correlation analysis between DEGs and differential expression metabolites also showed that levels of 5 DEGs (TRINITY_DN15939_c0_g1, TRINITY_DN20784_c0_g2, TRINITY_DN21962_c0_g2, TRINITY_DN23815_c1_g1, and TRINITY_DN26276_c0_g1) were positively correlated with all differential expression metabolites, while the other 6 DEGs (TRINITY_DN17945_c0_g2, TRINITY_DN21141_c0_g1, TRINITY_DN25788_c2_g8, TRINITY_DN22885_c0_g4, TRINITY_DN25099_c1_g5, and TRINITY_DN24108_c0_g2) were negatively correlated with all differential expression metabolites (Figure 6a, Supplementary Table S4) [37]. In addition, the combined metabolome and transcriptome analysis revealed a significant positive correlation between both *ANS* (TRINITY_DN15939_c0_g1) and *UFGT* (TRINITY_DN23815_c1_g1) (PCC > 0.82) and most metabolites (Figure 6b). These data indicated that expression patterns of *ANS* and *UFGT* have decisive roles in color development, and their regulatory relationships need to be further studied.

### 2.7. The Changes of Transcription Factor in the Process of Peel Coloring

Next, we focused on differentially expressed Transcription Factors (TFs) based on our transcriptome data. The MYB and bHLH TF families were the most abundant TF families during the fruit coloring process, followed by the B3, AP2/ERF, and NAC families (Table 1), many of these were expressed before samples began to show color. Additionally, the TFs were mostly up-regulated before the color change and down-regulated after the color change. Notably, the MYB and bHLH TF families are important transcription factors for anthocyanin transcriptional regulation, and 68% of bHLHs and 70% of MYB genes were down-regulated. Interestingly, we found that 2 MYB genes (TRINITY_DN26515_c0_g1 and TRINITY_DN21536_c0_g1) were significantly up-regulated after the fruit peel color changes, and their expression levels were consistent with the pattern of fruit peel pigment accumulation, indicating that they might be key regulators of fruit peel color.

### 2.8. Heterologous Expression of ChMYB1 in Apple Calli

**Figure 7:**
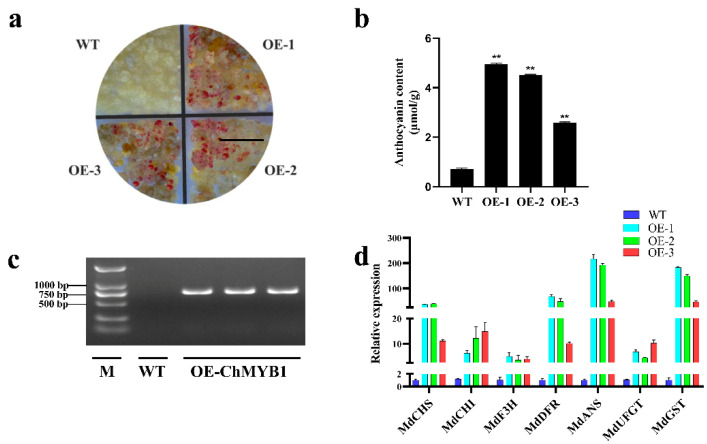
Overexpression of *ChMYB1* increases anthocyanin accumulation in apple calli. (**a**) Pigmentation phenotypes of flowers of untransformed calli (WT) and three *ChMYB1*-overexpressing apple calli lines (OE1, OE2, and OE3). (**b**) Anthocyanin content in *ChMYB1*-overexpressing apple calli. At least three independent experiments were performed for each sample, and a representative result is shown. Data are shown as the mean ± SD. (**c**) Confirmation of the presence of the transgene in OE-ChMYB1 calli by PCR analysis. M, 2000bp. (**d**) Expression profiles of anthocyanin biosynthesis-related genes (*MdCHS*, *MdCHI*, *MdF3H*, *MdDFR*, *MdANS*, *MdUFGT, and MdGST*) of WT, OE1, OE2, and OE3 in apple calli. ** above, the columns are significantly different at *p* < 0.05. Scale bar, 1 cm.

In order to understand the regulation of key transcription factors controlling fruit peel coloring, we selected the regulatory network of anthocyanin biosynthesis to verify its main contribution to pericarp coloring. Since MYB TF is the core regulator of anthocyanin biosynthetic pathway, we constructed an overexpression vector for *ChMYB1* (TRINITY_DN21536_c0_g1) to test its regulatory ability on anthocyanin biosynthesis. *Agrobacterium tumefaciens* strain GV3101 containing recombinant plasmid *35S:: ChMYB1* was heterologously transferred into apple calli (the ‘Orin’ apple cultivar (*M. domestica* Borkh.)) by a well-established apple tissue culture system, and three independent overexpression (OE) lines were obtained. After one week of light treatment, the color of apple calli of the three *ChMYB1* OE lines significantly turned red compared with the untransformed calli (WT), indicating that *ChMYB1* can affect the coloring of apple calli (Figure 7a). To characterize the phenotypes quantitatively, we measured the anthocyanin content of OE lines and the WT. It was found that the anthocyanin content of the OE lines was significantly higher than that of WT (Figure 7b). The presence of the transgene in ChMYB1 OE lines was confirmed by PCR (Figure 7c). The transcript levels of genes related to the anthocyanin biosynthetic pathway were analyzed by qRT-PCR. Compared with the WT, the expression of anthocyanin related *MdCHS*, *MdCHI*, *MdF3H*, *MdDFR*, *MdANS*, *MdUFGT,* and *MdGST* genes were significantly increased, which was consistent with the expression pattern of anthocyanin content (Figure 7d). Taken together, the heterologously expressed *ChMYB1* gene was involved in visible colorations of the calli by boosting the expression of anthocyanin biosynthesis genes.

## 3. Discussion

Fruit peel color is an important trait that effects the edible value of many fruits. Several recent reports have described key genes and enzymes for fruit peel color formation and anthocyanin accumulation in fruit trees, including pear and apple [16,29]. However, the regulation of fruit peel color is a complex process. Most of the previous studies analyzed the mechanism of fruit peel color formation by methods of physiology, cytology, and molecular biology, but the mechanism of fruit peel color regulation from the perspective of small molecular metabolites needs to be studied further. In addition, as far as we have known, the differences of metabolites of *C. humilis* peel color changes at different developmental stages have not been studied. Previous studies have shown that anthocyanins are related to the formation of red fruit [38]. In order to further reveal the molecular mechanism of anthocyanin biosynthesis in red fruit peel, based on transcriptome analysis and metabolic profiling, the changes of four developmental stages of fruit peel development of *C. humilis* were qualitatively and quantitatively analyzed. From this foundation, the regulation mechanism of fruit peel color was further studied by metabolite and gene expression analysis.

Metabolomics is an emerging omics technology that, similar to genomics and proteomics, can be used to qualify and quantify metabolites of small molecular weight within the cells of an organism [39]. Plant metabolomics is a new field in the post genomic era, and has been widely applied for the investigation of patterns of metabolite accumulation. Furthermore, this technology was used to reveal the change rule of genes and metabolites in various tissues. Using a targeted metabolomics approach, we detected 11 anthocyanin related metabolites with significant differences among developmental stages of fruit peel, all of which were increased. They were mainly divided into three categories: Pelargonidin, Cyanidin, and Peonidin. Notably, the levels of two anthocyanins, cyanidin *O*-syringic acid and pelargonidin 3-*O*-beta-d-glucoside (callitephin chloride), increased significantly during fruit peel development in *C. humilis*. Besides, Yi et al. [40] found that Cyanidin *O*-syringic acid is one of the main anthocyanin derivatives of red pericarp longan. Accordingly, we suggested that the accumulation of these anthocyanins accounted may be the reason for the red pigmentation of fruit peel during fruit development.

In the past ten years, the anthocyanin metabolism pathway has been basically clear in model plants, and the research on anthocyanin biosynthesis related genes has also made important progress in fruit crops [32,41]. However, most genes have been obtained and identified by traditional study techniques. Since, transcriptome analysis has been regarded as an important method to study the expression level, structure, and function of genes in order to reveal phenotypic characteristics. This enabled the search for useful information about the accumulation and regulation of anthocyanin in different developmental stages of *C. humilis* peel coloration. In this study, we found that most of upstream structural genes (*PAL* and *C4H*) in the early committed steps were highly active in young fruits but were gradually decreased with fruit peel development (Figure 5). This study also showed that *4CL* genes encode the same enzyme and have different expression patterns (Figure 5). For example, seven *4CL* genes were found in *C. paliurus* [42]. These findings suggest that different genes of the multiple gene family play different roles in the anthocyanin biosynthetic pathway. Further research is required to determine the role these genes play during the biosynthesis of specific anthocyanin. Previous studies indicated that anthocyanin biosynthesis involves multiple enzymes encoded by early step structural genes (*CHS*, *CHI*, and *F3H*) and anthocyanin-specific biosynthesis genes (*DFR*, *ANS*, and *UFGT*) [43]. In this study, the expression levels of *CHS*, *CHI*, *F3H*, *DFR*, *ANS* and *UFGT* increased significantly with the development of fruit peel. *UFGT*, which is involved in the last step of anthocyanin biosynthetic pathway, played an important role in anthocyanin metabolism, *UFGT* can glycosylate unstable anthocyanins, and glycosylation is essential to stabilize them and also serve as a signal for transport of the anthocyanins to vacuoles, where they can function as pigments [35]. Our results suggested that during the reddening of the fruit peel, the accumulation of anthocyanin metabolites is positively correlated with *UFGT* expression levels. *UFGT* genes (TRINITY_DN23815_c1_g1 and TRINITY_DN19893_c1_g5), which are involved in the synthesis and accumulation of anthocyanins, were gradually activated during ripening process of *C. humilis* fruit peel. Similarly, Steyn et al. [44] reported that *UFGT* activity increases in fruit development. Therefore, we proposed that *UFGT* during fruit ripening could potentially promote *C. humilis* peel coloring. These results identified *UFGT* genes that play an important role in the anthocyanin biosynthetic pathway in the peels of *C. humilis* at different developmental stages.

Metabolites can provide direct insight into biological processes and mechanisms [45]. In this study, we detected 16 metabolites, including 11 differential expression metabolites, providing reference values for the isolation and identification of functional compounds in fruit peel. Our combined metabolome and transcriptome analysis covered a significant positive correlation between the synthesis of two genes (*ANS*, TRINITY_DN15939_c0_g1; *UFGT*, TRINITY_DN23815_c1_g1) in the anthocyanin pathway (ko00942) and differential expression metabolites (Figure 6b), establishing a new interesting connection between relative anthocyanin related metabolites and genes. This study identified significant positive correlations between *ANS*, *UFGT* enzyme genes and metabolites, maybe major determinants of anthocyanin biosynthesis in fruit peel, thus further accounting for *C. humilis* fruit peel coloration.

Many studies confirmed that in addition to DEGs, TFs play a key role in the regulation of color changes and the activity of pigment biosynthetic pathways [39]. MYB TFs are key modulators of the structural genes in the anthocyanin biosynthetic pathways [26,39,46]. For example, *AcMYB110a* regulates anthocyanin production in petals by promoting the expression of *F3GT1* in *Actinidia* [47]. *McMYB10* plays an important role in ever-red leaf coloration by positively regulating *McF3′H* in crabapple [48]. *MdMYB308L* enhances its binding to the promoter of *MdDFR* by interacting with *MdbHLH33*, and then actively regulates the accumulation of anthocyanins in apple [29]. Consistent with these results, we identified two candidate *MYB* genes (TRINI-TY_DN26515_c0_g1 and TRINITY_DN21536_c0_g1) with the potential to positively regulate the anthocyanin biosynthetic genes in the RNA-Seq data, and their expression abundance was consistent with anthocyanin accumulation (Figure 2 and Figure 5a). In addition, we found that the expression pattern of *UFGT* (TRINITY_DN19893_c1_g5) and *MYB* genes (TRINI-TY_DN26515_c0_g1 and TRINITY_DN21536_c0_g1) was consistent, and *UFGT* was also positively correlated with anthocyanin accumulation. Reports have shown that *MdMYB90-like* activates anthocyanin biosynthesis by binding to the promoters of *CHS* and *UFGT* [49]. Therefore, these results indicate that the two MYB TFs may regulate the expression of the *UFGT* gene and thus contribute to the accumulation of anthocyanin. In addition, the overexpression of *ChMYB1* in apple calli promoted anthocyanin biosynthesis (Figure 7). Moreover, GST was reported to participate in the transport of anthocyanin [50], and *MdGST* is significantly up-regulated in the overexpression calli, which may play an important role in the transport of anthocyanins in transgenic apple calli. Thus we obtained the evidence that *ChMYB1* (TRINITY_DN21536_c0_g1) regulates anthocyanin biosynthesis, but its transcriptional regulation in anthocyanin biosynthesis of *C. humilis* fruit peel needs to be further studied.

## 4. Materials and Methods

### 4.1. Plant Materials

*C. humilis* (cv. Zhangwu) was grown at the Xiangyang Farm of Northeast Agricultural University in Harbin, Heilongjiang Province, China. Fruits samples were collected from 30, 42, 57, and 70 days post-anthesis (DPA), designated young fruit (S1), green fruit (S2), slightly red fruit (S3), and red fruit (S4). Three independent biological replicates were collected per stage, each with six fruits randomly from six *C. humilis* trees, every two of which were set as a biological replication. The peels were carefully excised with scalpels, collected the peel, frozen immediately in liquid nitrogen, and stored at −80 °C until use. The total anthocyanin content was measured using a UV1800 ultraviolet spectrophotometer (SHIMADZU, Japan) following the method described by Shen et al. [51].

### 4.2. Metabolome Analysis

The sample preparation, analysis, metabolite qualitative and quantification were carried out by MWDB (Metware Biotechnology Co., Ltd., Wuhan, China) according to their standard procedures [52,53,54]. The instrument system of metabolite data acquisition was mainly ultra-high performance liquid chromatography (HPLC) (shim pack UFLC Shimadzu cbm30a, http://www.shimadzu.com.cn/) which accessed on 10 December 2019, and tandem mass spectrometry (MS/MS) (Applied Biosystems 4500 qtrap, http://www.appliedbiosystems.com.cn/) which accessed on 10 December 2019, (Supplementary Table S5). The analytical conditions were as follows, HPLC: column, Waters ACQUITY UPLC HSS T3 C18 (1.8 µm, 2.1 mm*100 mm); solvent system, water (0.04% acetic acid): acetonitrile (0.04% acetic acid); gradient program,100:0*v*/*v* at 0 min, 5:95*v*/*v* at 11.0 min, 5:95*v*/*v* at 12.0 min, 95:5*v*/*v* at 12.1 min, 95:5*v*/*v* at 15.0 min; flow rate, 0.40 mL/min; temperature, 40 °C; injection volume: 5 μL. The effluent was alternatively connected to an ESI-triple quadrupole-linear ion trap (Q TRAP)-MS. The offline raw data after mass spectrometry analysis through the Software Analyst 1.6.3 was opened and browsed, and used for qualitative and quantitative analysis, the vertical for ion detection of ion flow intensity (CPS, count per second), the abscissa for the retention time of the detected metabolites (the Retention time, Rt) are shown in Figure S5. The data of metabolite content were normalized by range method and R software was used (www.r-project.org/) which accessed on 12 December 2019, and the accumulation patterns of metabolites among different samples were analyzed by hierarchical cluster analysis (HCA). The analysis of metabonomics data combines the methods of univariate and multivariate statistical analysis, and according to the characteristics of data, multi-perspective analysis was also carried out, and finally the differential expression metabolites were accurately mined.

### 4.3. RNA Isolation and Illumina Sequencing

Total RNA was extracted from four developmental stages of the fruit peel using the Omega RNA Extraction Kit (Biel, Switzerland). The 1% agarose gel electroassay was used to detect degradation and impurities. RNA quantity and quality were determined using a Keio K5500 spectrophotometer (Kaio, Beijing, China), Agilent Bioanalyzer 2100 system, and Agilent RNA 6000 Nano Kit (Agilent Technologies, Palo Alto, CA, USA). A total of 4 g of total RNA per sample (12 samples = 4 stages × 3 biological replicates) was used as input material for RNA-seq library preparation (Annoroad Biotechnology, Zhejiang, China). Trinity was for robust de novo assembly. Raw data (in FASTq format) were processed using Perl scripts to measure Q30 values and GC contents. After removing low-quality reads, the remaining clean data were used for all downstream analyses. TransDecoder identifies candidate coding regions within transcript sequences, such as those generated by de novo RNA-Seq transcript assembled by Trinity. The identification of an ORF provides the first evidence to prove that a new sequence of DNA being a gene encoding for a particular protein. Trinotate was used for performing the functional annotation of unigenes and ORFs. Trinotate is a comprehensive annotation suite designed for automatic functional annotation of transcriptomes, particularly for de novo assembled transcriptomes, from model to non-model organisms. Trinotate makes use of a number of different well referenced methods for functional annotation including homology search to known sequence data (BLAST+/SwissProt), protein domain identification (HMMER/PFAM), protein signal peptide and transmembrane domain prediction (singalP/tmHMM), and comparison to currently current annotation databases (EMBL Uniprot eggNOG/GO Pathways databases) [52].

### 4.4. Transcriptome Data Analysis

Differential expression analysis was performed using DESeq2 v1.4.5 according to the standard procedures of Annoroad Biotechnology, the corrected *p* < 0.05 and |log2Ratio| ≥ 1 was used to identify the differentially expressed genes (DEGs), using gene ontology (GO) and Kyoto Encyclopedia of Genes and Genomes (KEGG) enrichment analyses of differentially expressed genes.

### 4.5. QRT-PCR and Expression Validation

Based on the results of the transcriptomic analysis, 12 anthocyanin biosynthetic genes and 2 MYB genes were chosen for verification by qRT-PCR. The qRT-PCR reactions were conducted using UltraSYBR Mixture (CWBIO, Beijing, China) with the Roche Light Cycler 480 II real-time PCR System (Roche, Basel, Switzerland) according to the manufacturer’s instructions. Values were presented as means of three biological replicates and three technical replicates. The relative gene expression levels were calculated by the 2^−ΔΔCt^ method; the relative transcript levels were calculated and normalized to *Actin* transcript levels [38]. Primers were design by online website IDT (https://sg.idtdna.com/Scitools/Applications/RealTimePCR/) which accessed on 27 March 2021. All primers used herein are listed in Table S6.

### 4.6. Molecular Cloning of ChMYB1

Total RNA from *C. humilis* red fruit peel was isolated using the Omega RNA Extraction Kit (Biel, Switzerland) in accordance with the manufacturer’s protocol. Approximately 0.2 μg of cDNA was synthesized using the PrimeScript^TM^ RT reagent Kit with gDNA Eraser (TaKaRa, Dalian, China) and was then used as the template for subsequent PCR amplification. Based on the transcriptome database of *C. humilis* acid bacteria constructed by our research team, *ChMYB1* was isolated by using a pair of specific primers (Supplementary Table S6).

### 4.7. Transformation of Apple Calli

ChMYB1 (TRINITY_DN21536_c0_g1) was cloned into the GV1300 vector and then transformed into Agrobacterium tumefaciens GV3101. As for the transformation of apple calli, 16-day-old untransformed calli were co-cultured for 20 min with Agrobacterium carrying ChMYB1-GV1300, and the apple calli were co-cultured on MS medium supplementing 0.5 mgL^−1^ IAA and 1.5 mgL^−1^ 6-BA for two days at 24 °C. Then, the apple calli were washed three times with sterile water and transferred to selective media supplementing 40 mgL^−1^ hygromycin. Apple calli were grown at 24 °C under dark conditions for 15 days, and then continuous light for 7 days. Transformation of apple calli (the ‘Orin’ apple cultivar (M. domestica Borkh.)) was performed as described by Li et al. [55]. The calli were sampled after treatment, frozen in liquid nitrogen, and stored at −80 °C until use. RNA extraction and reverse transcription were performed using the Omega RNA Extraction Kit (Biel, Switzerland) and the PrimeScript^TM^ RT reagent Kit with gDNA Eraser (TaKaRa, Dalian, China), respectively. The primers used for qRT-PCR are listed in Table S6.

### 4.8. Statistical Methods

According to the data of anthocyanin-related gene expression level and metabolite content, the correlation between gene expression level and metabolite content was determined by selecting the detection correlation with P value ≤ 0.05. In addition, the networks were visualized using Cytoscape. All statistical analyses were conducted by SPSS 17.0 software.

## 5. Conclusions

Collectively, the mechanisms underlying anthocyanin accumulation in *C. humilis* fruit peel were analyzed by MRM, transcriptomics, and qRT-PCR. At least 16 anthocyanins exhibited differences among developmental stages, and cyanidin *O*-syringic acid and pelargonidin 3-*O*-beta-d-glucoside (callitephin chloride) were identified as the major anthocyanins. Moreover, the anthocyanin biosynthetic genes were identified and analyzed by transcriptome data and qRT-PCR. These results indicated that *ANS* and *UFGT* were the key genes involved in anthocyanin accumulation. Meanwhile, two MYB genes (TRINITY_DN26515_c0_g1 and TRINITY_DN21536_c0_g1) participated in anthocyanin synthesis pathway and potentially regulated the expression of related genes in *C. humilis* fruit peel. Moreover, the overexpression of *ChMYB1* (TRINITY_DN21536_c0_g1) in apple calli could be involved in regulating the coloring of fruits. It was also found that *UFGT* (TRINITY_DN19893_c1_g5) and *ChMYB1* (TRINI-TY_DN21536_c0_g1) have similar expression patterns. This study improves our understanding of anthocyanin accumulation and the molecular mechanism underlying anthocyanin biosynthesis in *C. humilis* fruit peel. However, the precise mechanism requires further studies.

## Figures and Tables

**Table 1:** Differentially expressed transcription factors (TFs) in fruit peel.

DEG Set	TF Family	Number of DEGs	Up	Down	Description
S2_S1	ARF/ERF	20	12	8	Ethylene-responsive transcription factor
	B3	12	7	5	B3 DNA-binding domain
	bHLH	22	11	11	basic helix-loop helix
	bzip	8	5	3	Basic region leucine zipper
	C2H2	8	5	3	Zinc finger C2H2 domain-containing protein
	C3H	9	7	2	Zinc finger CCCH domain-containing protein
	DBB	8	7	1	double B-box zinc finger protein
	G2-like	11	5	6	Golden2-Like protein
	GRAS	8	7	1	C-terminal GRAS domain
	HSF	22	18	4	Heat stress transcription factor
	MYB	28	20	8	MYB-related protein
	NAC	15	14	1	NAC domain-containing protein
	S1Fa-like	12	6	6	Leucine-rich repeat protein
	other TFs	104	56	48	
	total	287	180	107	
S3_S2	ARF/ERF	98	24	74	Ethylene-responsive transcription factor
	B3	121	27	94	B3 DNA-binding domain
	bHLH	152	53	99	basic helix-loop helix
	bzip	62	25	37	Basic region leucine zipper
	C2H2	76	27	49	Zinc finger C2H2 domain-containing protein
	C3H	52	17	35	Zinc finger CCCH domain-containing protein
	DBB	17	3	14	double B-box zinc finger protein
	G2-like	67	22	45	Golden2-Like protein
	GRAS	47	14	33	C-terminal GRAS domain
	HSF	64	14	50	Heat stress transcription factor
	MYB	159	48	111	MYB-related protein
	NAC	92	25	67	NAC domain-containing protein
	S1Fa-like	32	7	25	Leucine-rich repeat protein
	other TFs	717	252	465	
	total	1756	558	1198	
S4_S3	ARF/ERF	78	25	53	Ethylene-responsive transcription factor
	B3	91	32	59	B3 DNA-binding domain
	bHLH	116	33	83	basic helix-loop helix
	bzip	55	17	38	Basic region leucine zipper
	C2H2	47	13	34	Zinc finger C2H2 domain-containing protein
	C3H	39	13	26	Zinc finger CCCH domain-containing protein
	DBB	6	3	3	double B-box zinc finger protein
	G2-like	40	17	23	Golden2-Like protein
	GRAS	30	15	15	C-terminal GRAS domain
	HSF	39	25	14	Heat stress transcription factor
	MYB	112	32	80	MYB-related protein
	NAC	74	32	42	NAC domain-containing protein
	S1Fa-like	43	4	39	Leucine-rich repeat protein
	other TFs	484	160	324	
	total	1254	421	833	

## Data Availability

All data generated or analyzed during this study are included in this published article and its Supplementary information files. The raw RNA-seq data are freely available at: www.ncbi.nlm.nih.gov/bioproject/PRJNA636897 which accessed on 3 June 2020.

## Supplementary Materials

The following are available online at https://www.mdpi.com/article/10.3390/ijms222111880/s1.
