# Supplementary material for: Integrated Analysis of the Metabolome and Transcriptome on Anthocyanin Biosynthesis in Four Developmental Stages of Cerasus humilis Peel Coloration
Source: Int J Mol Sci. 2021 Nov 2;22(21):11880. doi: 10.3390/ijms222111880 (PMC8585068; doi:10.3390/ijms222111880)
Supplement: Supplementary file 1 [file ijms-22-11880-s001.zip › Supplementary Figures.pdf]

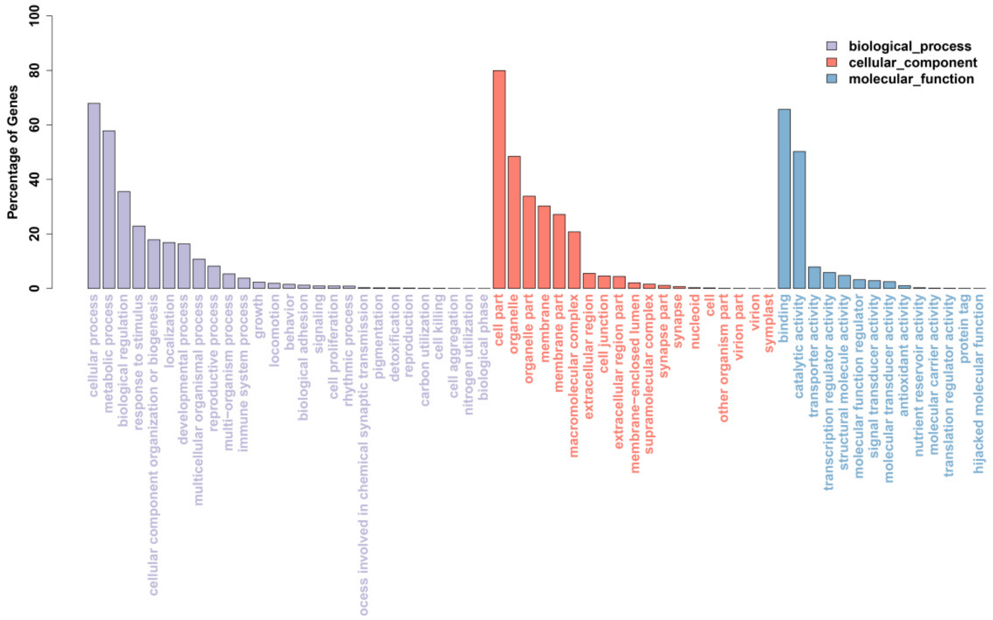

Figure S1. GO classification of unigenes from *Cerasus humilis* fruit peel.

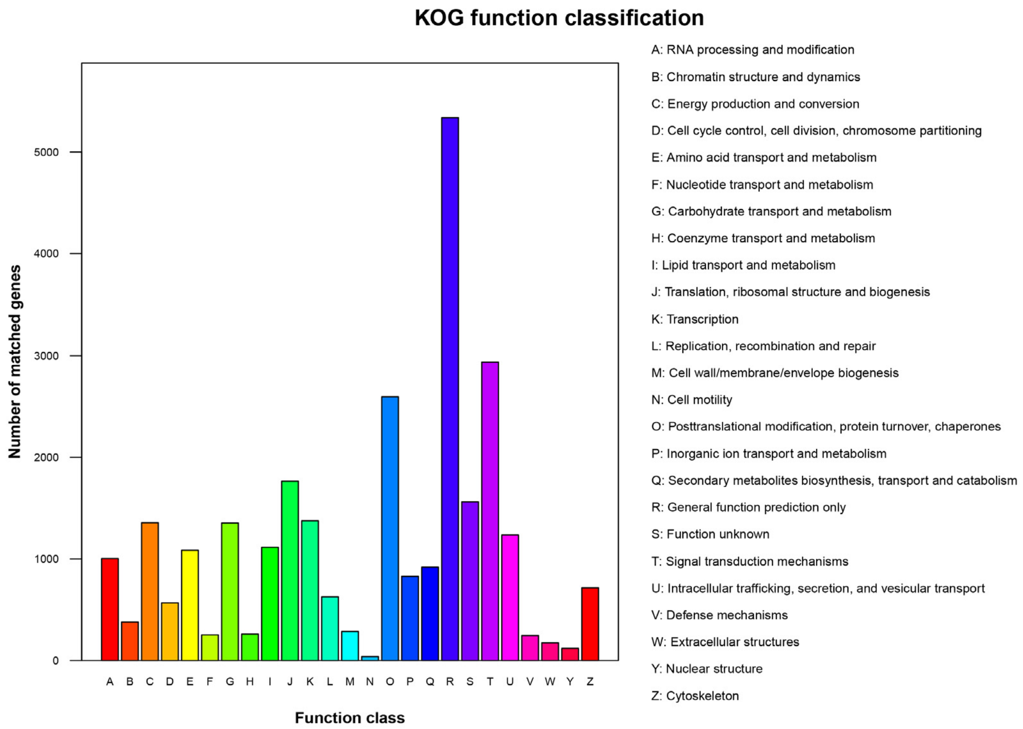

Figure S2. Classification of unigenes from *Cerasus humilis* fruit peel.

a

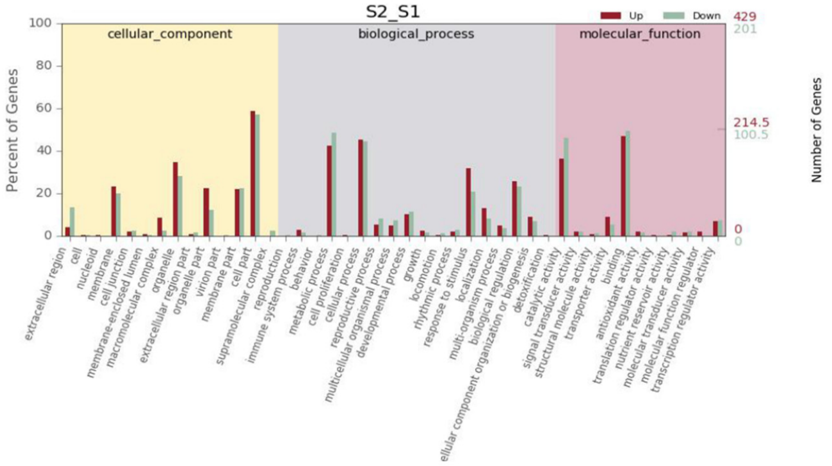

b

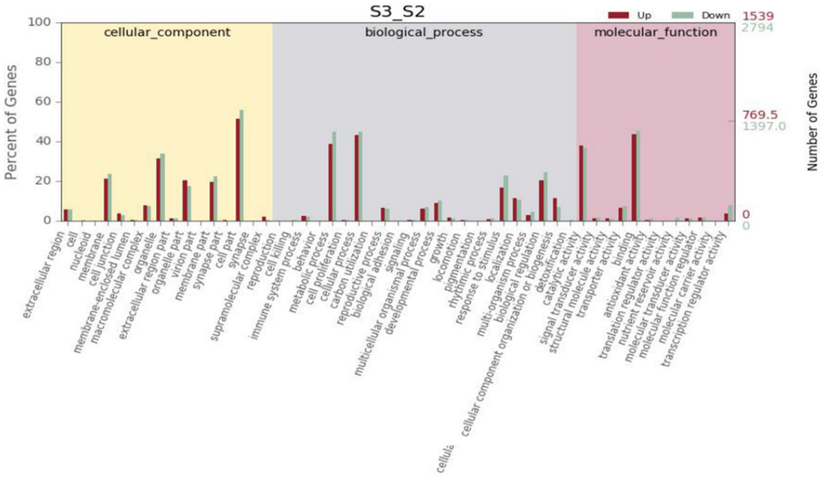

c

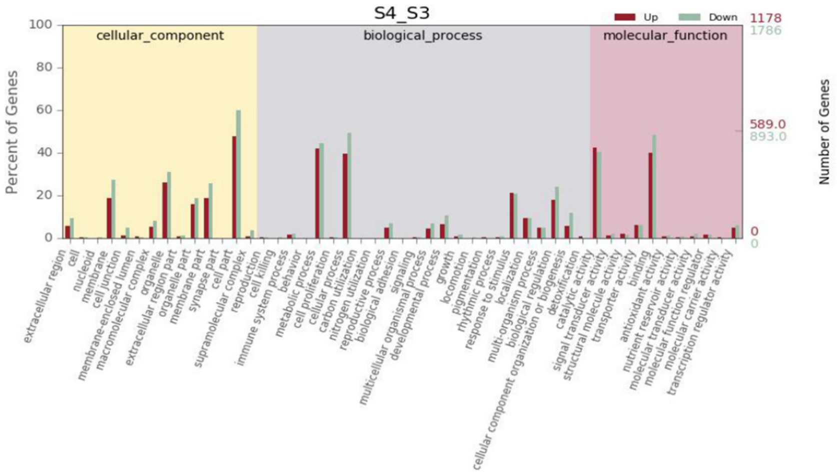

Figure S3. Functional categorization of genes with significant transcriptional changes the differental stages of fruit peel. S2_S1(a), S3_S2 (b) and S4_S3 (c). red indicates up-regulated and gray indicated down-regulated genes.

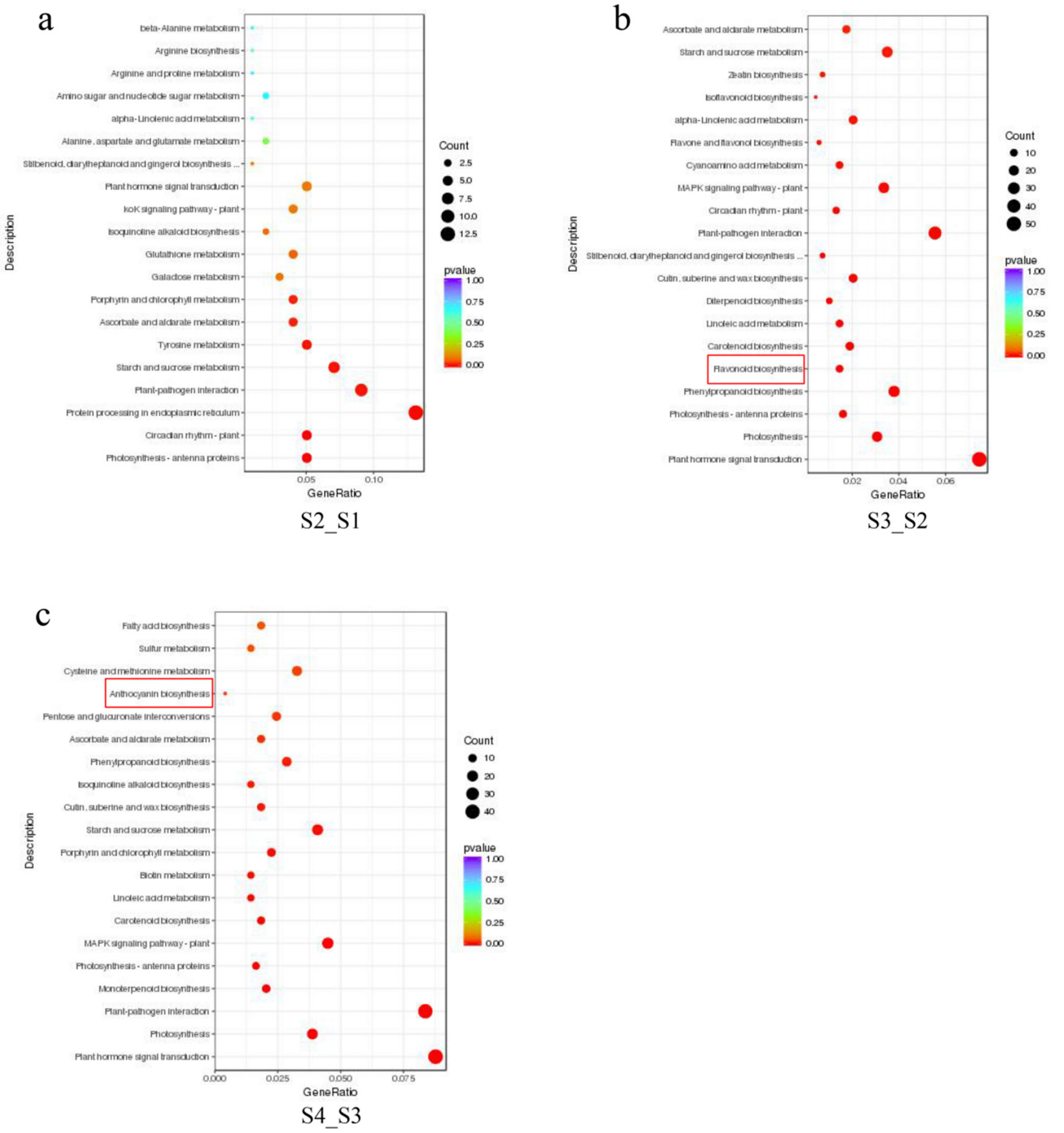

Figure S4. Scatterplot of the top 20 enriched KEGG pathway statistics in DEGs. S2_S1(a), S3_S2 (b) and S4_S3 (c). The q value is the multiple hypothesis test-corrected P value. The q value ranges from [0-1]. The closer that number is to 0, the more significant the enrichment is. The greater the rich factor, the greater the degree of enrichment is.

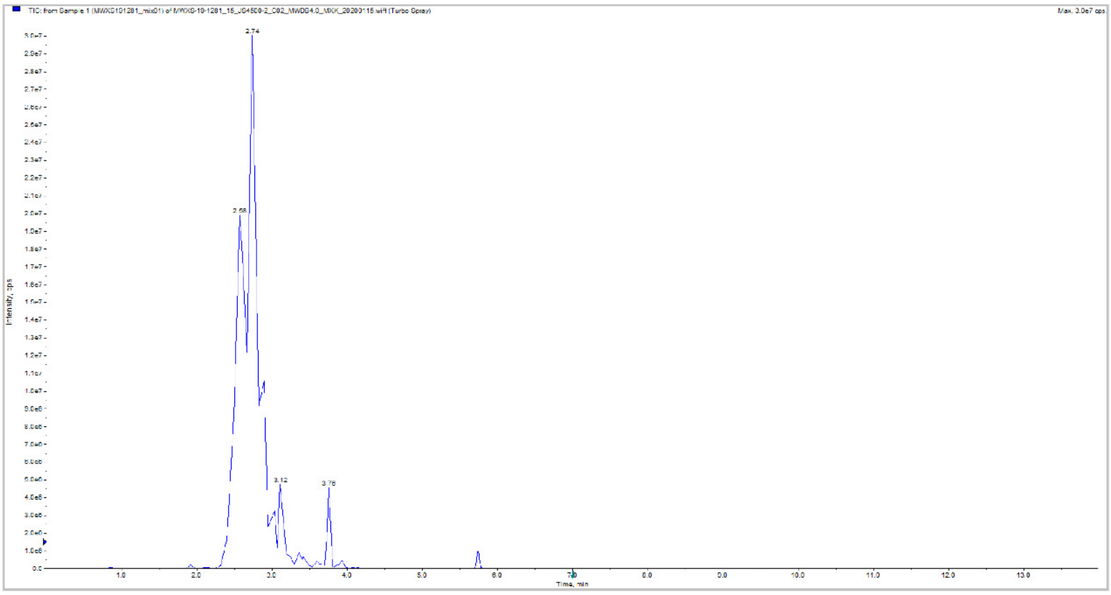

Figure S5. The total ion current (TIC) of *Cerasus humilis* fruit peel.
